# High-Throughput *flaA* Short Variable Region Sequencing to Assess *Campylobacter* Diversity in Fecal Samples From Birds

**DOI:** 10.3389/fmicb.2018.02201

**Published:** 2018-09-25

**Authors:** Qian Zhang, Gabriel A. Al-Ghalith, Mayumi Kobayashi, Takahiro Segawa, Mitsuto Maeda, Satoshi Okabe, Dan Knights, Satoshi Ishii

**Affiliations:** ^1^BioTechnology Institute, University of Minnesota, St. Paul, MN, United States; ^2^Bioinformatics and Computational Biology, University of Minnesota, Minneapolis, MN, United States; ^3^Division of Environmental Engineering, Graduate School of Engineering, Hokkaido University, Sapporo, Japan; ^4^Center for Life Science Research, University of Yamanashi, Yamanashi, Japan; ^5^National Institute of Polar Research, Tokyo, Japan; ^6^Department of Computer Science and Engineering, University of Minnesota, Minneapolis, MN, United States; ^7^Department of Soil, Water, and Climate, University of Minnesota, St. Paul, MN, United States

**Keywords:** *Campylobacter*, diversity, pathogen, source tracking, genotyping, *flaA*, next generation sequencing

## Abstract

Current approach to identify sources of human pathogens is largely dependent on the cultivation and isolation of target bacteria. For rapid pathogen source identification, culture-independent strain typing method is necessary. In this study, we designed new primer set that broadly covers *flaA* short variable region (SVR) of various *Campylobacter* species, and applied the *flaA* SVR sequencing method to examine the diversity of *Campylobacter* spp. in geese fecal samples (*n* = 16) with and without bacteria cultivation. Twenty-three *Campylobacter* strains isolated from the 16 geese fecal samples were grouped similarly by conventional *flaA* restriction fragment length polymorphism (RFLP) method and by the *flaA* SVR sequencing method, but higher discriminant power was observed in the *flaA* SVR sequencing approach. For culture-independent *flaA* SVR sequencing analysis, we developed and optimized the sequence data analysis pipeline to identify as many genotypes as possible, while minimizing the detection of genotypes generated by sequencing errors. By using this pipeline, 51,629 high-quality *flaA* sequence reads were clustered into 16 operational taxonomic units (=genotypes) by using 98% sequence similarity and >50 sequence duplicates. Almost all *flaA* genotypes obtained by culture-dependent method were also identified by culture-independent *flaA* SVR MiSeq sequencing method. In addition, more *flaA* genotypes were identified probably due to high throughput nature of the MiSeq sequencing. These results suggest that the *flaA* SVR sequencing could be used to analyze the diversity of *Campylobacter* spp. without bacteria isolation. This method is promising to rapidly identify potential sources of *Campylobacter* pathogens.

## Introduction

*Campylobacter* species are one of the most common bacterial foodborne pathogens, causing gastrointestinal disorders such as bloody diarrhea, dysentery syndrome, and cramps (Silva et al., 2011). *Campylobacter* is estimated to cause more than two million illnesses, 13,000 hospitalizations, and >100 deaths each year in the United States (Silva et al., 2011), and the incidence of *Campylobacter* illness has been increased by 13% since 2006–2008 (Crim et al., 2015). The main source of the *Campylobacter* infection is the consumption of contaminated water and foods, especially poultry products (Silva et al., 2011).

Although the most common hosts for *Campylobacter* spp. are avian species, including commercial poultry species (e.g., chickens and turkeys) and waterfowl (e.g., geese; Pacha et al., 1988), they are also found in other animals such as cattle, pigs, and dairy cows (Westrell et al., 2009; Silva et al., 2011). *Campylobacter* species cannot grow but can survive well in various environments such as fresh and coastal waters and sediment after they are released from their primary hosts (e.g., birds and animals). Thus, the environments can act as self-perpetuating reservoir of *Campylobacter* infection cycling between humans, wildlife, and domestic animals (Jones, 2001). In fact, *Campylobacter* spp. is frequently and abundantly detected in various environmental water samples (Ishii et al., 2014a; Oster et al., 2014; Zhang et al., 2016).

To protect human health, it is important to monitor levels of *Campylobacter* in food and water samples, and identify the sources of pathogens. While pathogen monitoring can be done by culture-independent quantitative PCR (qPCR) method (Ishii et al., 2013; Banting et al., 2016; Zhang et al., 2016), current pathogen source identification methods largely depend cultivation and isolation of pathogens. Various genotyping methods have been used to identify sources of *Campylobacter* strains, including pulsed-field gel electrophoresis (PFGE), restriction fragment length polymorphism (RFLP), multilocus sequence typing (MLST), antimicrobial resistance profiling, the CRISPR1 typing, comparative genomic fingerprinting, and *flaA* short variable region (SVR) sequencing (Taboada et al., 2013; Duarte et al., 2016; Calleros et al., 2017). Although these methods are useful in discriminating different *Campylobacter* strains, some of them require time and labor, while others do not have enough discriminating power. In addition, all methods are designed to characterize isolated *Campylobacter* strains, which is time and labor intensive (On, 2013). Sometimes *Campylobacter* cells enter viable-but-non-culturable (VBNC) state and become unavailable for isolation (Tholozan et al., 1999). Therefore, for rapid and comprehensive identification of *Campylobacter* sources, culture-independent strain typing method is desired.

Here, we propose to use culture-independent *flaA* SVR sequencing to rapidly identify *Campylobacter* genotypes in environmental samples. The objectives of this study were to (1) design new primer set that broadly covers *flaA* SVR of various *Campylobacter* species, (2) compare strain discrimination obtained by the *flaA* SVR sequencing done with the new primers with those obtained by a commonly used *flaA* RFLP method, and (3) apply the *flaA* SVR sequencing to examine the diversity of *Campylobacter* spp. in geese fecal samples without bacteria cultivation.

## Materials and Methods

### Geese Fecal Samples

Fecal samples from greater white-fronted goose (*Anser albifrons*) were collected from agricultural fields near Lake Miyajimanuma, Hokkaido, Japan, in September and October 2013. Details of the sampling location are described elsewhere (Ishii et al., 2014a). The collected geese fecal samples (*n* = 77) were stored in Whirl-Pak bags and kept on the ice during the transportation (<2 h). DNA was directly extracted from 0.2 g of geese fecal samples by using a QIAamp DNA Stool Mini kit (Qiagen). DNA samples were stored at −20°C until use.

### *Campylobacter* Strains

*Campylobacter* sp. strains were isolated from geese fecal samples as described below. One gram of fecal sample was added to 9 ml phosphate-buffered saline (PBS) gelatin buffer in a 15-ml centrifuge tube (Hamilton et al., 2010) and vigorously mixed for 30–45 s; 1 ml of the fecal suspension was transferred to 9 ml of Bolton broth (Oxoid) supplemented with laked horse blood (Remel) and antibiotics (20 μg/ml cefoperazone, 20 μg/ml trimethoprim, 20 μg/ml vancomycin, and 50 μg/ml cyclohexamide). The tubes were placed in microaerobic conditions created using AnaeroPack MicroAero (Mitsubishi Gas Chemical), and incubated at 37°C for 4 h followed by 42°C for 44 h. After incubation, a loopful culture (*ca*. 10 μl) was streaked onto modified *Campylobacter* blood-free agar (m-CCDA) medium (Oxoid) supplemented with 32 μg/ml cefoperazone and 10 μg/ml amphotericin B, and incubated under microaerobic conditions at 42°C for 48 h. Colonies formed on the m-CCDA medium were re-streaked on a new m-CCDA medium, and incubated under microaerobic conditions at 42°C for 48 h.

Colonies were confirmed as being *Campylobacter* species by sequencing the 16S rRNA gene sequences. In brief, DNA were extracted from well-isolated single colonies formed on the m-CCDA medium, by heating cells in 100 μl 0.05 M NaOH at 95°C for 15 min. After centrifugation, the supernatant was diluted 10-fold in MilliQ water, and used for PCR done with C412F and C1228R primers (Linton et al., 1996) as described previously (Ishii et al., 2006). PCR products were purified and sequenced as described previously (Ishii et al., 2016). *Campylobacter jejuni* JCM 2013, *Campylobacter coli* JCM 2529^T^, and *Campylobacter lari* JCM 14870^T^ were used for quality control of growth media and PCR.

### *flaA* RFLP

The *flaA* RFLP was performed to genotype *Campylobacter* strains. The *flaA* fragments (*ca*. 1,800 bp) were PCR-amplified using flaA-F (5′-ATGGGATTTCGTATTAACAC-3′) and flaA-R (5′-CTGTAGTAATCTTAAAACATTTTG-3′) primers (Wassenaar and Newell, 2000). The PCR reaction mixture (20 μl) contained 1 × *Ex Taq* buffer (Takara Bio), 0.2 μM of each primer, 0.2 mM of each dNTP, 0.4 U of *Ex Taq* DNA polymerase (Takara Bio), and 1 μl of DNA template. PCR was performed using a Veriti 96-well thermal cycler (ThermoFisher Scientific) with the following conditions: initial annealing at 95°C for 5 min, followed by 35 cycles of 95°C for 30 s, 55°C for 30 s, and 72°C for 2 min. After final extension at 72°C for 7 min, the PCR mixtures were stored at 4°C.

The PCR products (7 μl) were individually digested with *Dde* I and *Hinf* I (Takara Bio) according to the manufacturer’s instruction. Digested fragments were separated by 1.5% agarose gel electrophoresis. Gels were stained with GelRed (Biotium, Hayward, United States) and visualized under UV light. Sizes of the DNA fragments were calculated based on the external size standards (i.e., 100-bp DNA ladder) by using BioNumerics software ver. 6.6. Dendrogram was constructed using Jaccard similarity and the unweighted pair group method with arithmetic means (UPGMA) clustering method.

### *flaA* SVR Sequencing for Pure Culture Strains

A new primer set was designed using DegePrime (Hugerth et al., 2014) to broadly cover *flaA* SVR of various *Campylobacter* species. About 400-bp *flaA* fragment was amplified using Campy_flaA_235F (5′-GATAARGCWATGGATGAGCA-3′) and Campy_flaA_635R (5′-CHGTYCCWACWGAAGTWGAA-3′; **Supplementary Figure S1**). The PCR reaction mixture (20 μl) contained 1 × *Ex Taq* buffer (Takara Bio), 0.5 μM of each primer, 0.2 mM of each dNTP, 0.4 U of *Ex Taq* DNA polymerase (Takara Bio), and 1 μl of DNA template. PCR was performed using a Veriti 96-well thermal cycler with the following conditions: initial annealing at 95°C for 5 min, followed by 35 cycles of 95°C for 30 s, 52°C for 30 s, and 72°C for 30 s. After final extension at 72°C for 7 min, the PCR mixtures were stored at 4°C. The PCR products were purified using the FastGene Gel/PCR Purification Kit (Nippon Genetics) and sequenced bidirectionally using the BigDye Terminator v3.1 Cycle Sequencing Kit (ThermoFisher Scientific) and ABI 3730*xl* capillary sequencer (ThermoFisher Scientific). The resulting nucleotide sequences were assembled using Phred-Phrap program (Ewing et al., 1998).

### *flaA* SVR Sequencing for Environmental Samples

To sequence *flaA* SVR from environmental samples, MiSeq sequence library was prepared. In brief, *flaA* SVR was amplified using the *flaA*-specific primers tagged with Illumina overhang adaptor sequences: Campy_flaA_235F_adaptor, 5′-tcgtcggcagcgtcagatgtgtataagagacagGATAARGCWATGGATGAGCA-3′ and Campy_flaA_635R_adaptor, 5′-gtctcgtgggctcggagatgtgtataagagacagCHGTYCCWACWGAAGTWGAA-3′ where Illumina overhang adaptor sequences are shown in lower case. Optimal number of PCR cycles was determined by qPCR. The qPCR reaction mixture (10 μl) contained 1 × SYBR Premix *Ex Taq* (Tli RNase H plus) ROX plus (Takara Bio), 0.4 μM of each primer, and 1 μl of DNA template. PCR was performed using a StepOnePlus Real-Time PCR System (ThermoFisher Scientific) with the following conditions: initial annealing at 95°C for 30 s, followed by 30–40 cycles of 95°C for 5 s, 58°C for 30 s, and 72°C for 30 s. The cycle numbers were set to reach the log-linear phase of the PCR kinetics based on the qPCR results to minimize PCR-dependent biases during amplification (Ishii et al., 2014b). The PCR products were purified using the LaboPass PCR Kit (Cosmo Genetech).

The purified PCR products were tagged with sample-unique index and Illumina adapter sequences at their 5′ end (Nextera XT index Kit v2; Illumina) by PCR. The PCR reaction mixture (25 μl) contained 1 × KAPA HiFi HS ReadyMix (Kapa Biosystems), 2.5 μl each forward and reverse primers, and 2.5 μl of the purified first PCR products. The PCR was performed under the following conditions: 95°C for 3 min, 8 cycles of 95°C for 30 s, 55°C for 30 s, and 72°C for 30 s, and 72°C for 5 min. After agarose gel electrophoresis, PCR products (*ca*. 500 bp) were excised from the gel and purified using the FastGene Gel/PCR Purification Kit. Tagged amplicons were pooled and 8 pmol of the pooled amplicons were sequenced by Illumina MiSeq platform in 300 bp paired-end sequencing reaction with v3 reagent kit (Illumina) according to the manufacturer’s instruction. PhiX Control v3 (Illumina) was added at 20% of the input DNA amount as a quality control for the MiSeq run.

### Analysis of *flaA* SVR Sequencing

The raw MiSeq sequencing reads were processed with the SHI7 program (Al-Ghalith et al., 2018). The scripts are available at GitHub^1^. In brief, Illumina adaptor and primer sequences were removed, and paired-end sequences were assembled. The resulting sequences were further filtered by size of the expected *flaA* amplicon length (355–370 bp) and by removing singletons. Sequence denoising was done using two parameters: number of duplicated reads (*D*) and the sequence alignment similarity (*S*) of operational taxonomic units (OTUs). Specifically, we systematically varied *D* (1, 2, 3, 5, 10, 25, 50, and 100 reads) and *S* values (97, 97.25, 97.5, 97.75, 98, 98.25, 98.5, 98.75, and 99%) to identify the largest number of duplicates and the highest sequence alignment similarity to cluster sequences to OTUs that represent >95% of all length-trimmed amplicon data. In this study, optimizing this criterion led to the selection of 98% alignment similarity and 50 duplicates. These optimized parameters were used to align all of the trimmed amplicon data to the representative set with the exhaustive BURST aligner (Al-Ghalith and Knights, 2017).

The *flaA* nucleotide sequences were aligned using ClustalW, and used to generate a phylogenetic tree. Phylogenetic trees were constructed based on the maximum likelihood, maximum parsimony, and neighbor-joining methods by using MEGA ver. 6.06 (Tamura et al., 2013).

### Nucleotide Sequence Accession Numbers

The *flaA* sequences of *Campylobacter* strains identified in this study were deposited to DDBJ/Emble/Genbank databases under accession numbers MG694324-MG694346. MiSeq data were deposited to DDBJ/Emble/Genbank Short Read Archive (SRA) under accession number SRP127268.

## Results

### Comparison Between *flaA* RFLP and *flaA* SVR Sequencing

**FIGURE 1:**
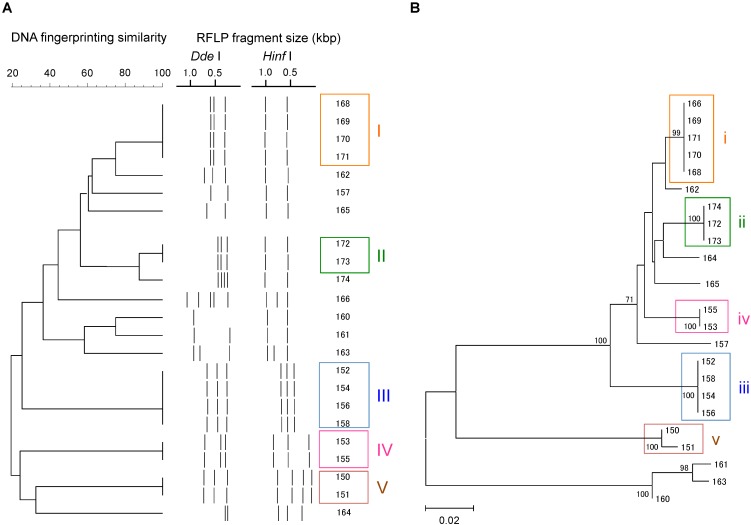
Clustering patterns of the *Campylobacter* strains isolated in this study, based on **(A)** *flaA* RFLP and **(B)** *flaA* SVR sequencing. Clustering was done using UPGMA method for *flaA* RFLP, while maximum likelihood method was used for *flaA* SVR sequencing.

A total of 23 *Campylobacter* strains were isolated from 16 geese fecal samples, and were characterized by *flaA* RFLP and *flaA* SVR sequencing. The *Campylobacter* strains were grouped similarly by the two methods (**Figure 1**). Based on the *flaA* RFLP and *flaA* SVR sequencing, 23 *Campylobacter* strains were clustered to 14 and 13 groups, respectively. When excluding singletons, five and four groups were formed by the *flaA* RFLP and *flaA* SVR sequencing, respectively. Same strains were grouped together by both methods, except Strains 13.166 and 13.174. While Strain 13.166 was a member of Group (i) by *flaA* SVR sequencing, this strain was clustered differently by the *flaA* RFLP. Similarly, Strain 13.174 was grouped differently between the *flaA* SVR sequencing and the *flaA* RFLP. This is probably due to the insufficient fragment digestion by restriction endonucleases. When we summed the size of the digested fragments, it exceeds the size of the original PCR product (*ca*. 1.8 kbp) in both Strains 13.166 and 13.174, suggesting the occurrence of insufficient fragment digestion. The same results were obtained when we repeated the restriction digestion with extended incubation. To verify the enzyme recognition sites, we sequenced the entire 1.8 kbp fragments of *flaA* of Strains 13.166 and 13.174, and digested *in silico* by using NEBcutter V2.0 software^2^. Based on the *in silico flaA* RFLP done with the sequenced *flaA* data (**Supplementary Figure S2**), Strains 13.166 and 13.172 were confirmed to belong to Group (i) and (ii), respectively, confirming that the different results obtained by the two methods were due to insufficient fragment digestion during the RFLP experiment.

We also observed that some strains that were indistinguishable by *flaA* RFLP could be separated by *flaA* SVR sequencing. For example, Strains 13.150 and 13.151 were 100% identical based on the *flaA* RFLP, although these strains were distinguished by *flaA* SVR. This is probably due to the presence of base mismatches in the *flaA* SVR region that could not be recognized by the restriction endonucleases, suggesting the higher discriminant power of *flaA* SVR than *flaA* RFLP.

### Quantity of *flaA* in Geese Samples

By using SYBR Green assay, we could quantitatively detect *flaA* in environmental samples. The PCR efficiency was 66.7% with a linear dynamic range from 20 to 2 × 10^6^ copies/μl. We detected *flaA* in 18.2% of geese fecal samples (14 out of 77 samples), with average density of 4.06 log_10_ copies/g feces. Sixteen out of 77 samples were positive for *Campylobacter* isolates; however, only five out of these 16 samples (31.3%) were positive for *flaA* qPCR (**Supplementary Table S1**). Nine samples were negative for *Campylobacter* isolates, but were positive for *flaA* qPCR. Densities of *Campylobacter* in isolate-positive and -negative fecal samples were 4.07 and 4.05 log_10_ copies/g feces and were not significantly different. Quantification limit of *flaA* in fecal samples was 3 log_10_ copies/g feces.

### *Campylobacter* Diversity Assessed by High-Throughput *flaA* SVR Sequencing

**FIGURE 2:**
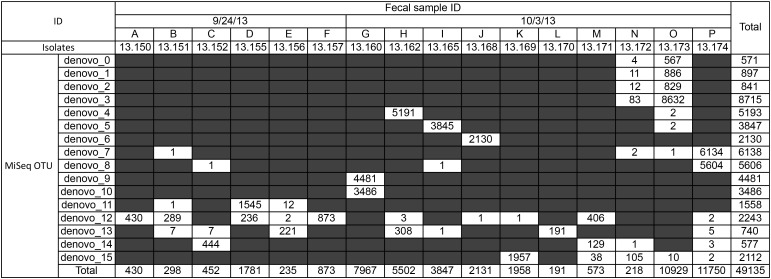
Number of sequence reads for each *flaA* operational taxonomic units (OTUs) in each fecal sample. Sequences were obtained by MiSeq analysis, and clusterd into OTU by using the SHI7 program (Al-Ghalith et al., 2018). Strain ID isolated from each fecal sample is also shown.

Our *flaA* SVR sequencing done with MiSeq identified great diversity of *Campylobacter* spp. in geese fecal samples. More than 16.6 M reads/run were obtained, but only sequences from the 16 geese fecal samples were used in this study. A total of 51,629 high-quality sequences with average Phred quality score of 37 and sequence length 355–370 bp were obtained from 16 geese fecal samples. By using our newly developed sequence data analysis pipeline, 49,135 out of 51,629 sequences (95.2%) were clustered into 16 OTUs. Most of these OTUs (81%; 13 out of 16 OTUs) were commonly detected in multiple fecal samples (**Figure 2**). Some of them (e.g., OTUs denovo_7, denovo_12, denovo_13, and denovo_14) appeared in multiple fecal samples collected at different sampling dates (September 24th and October 7th, 2013), suggesting that these *flaA* genotypes were common and indigenous member of geese gut flora.

**FIGURE 3:**
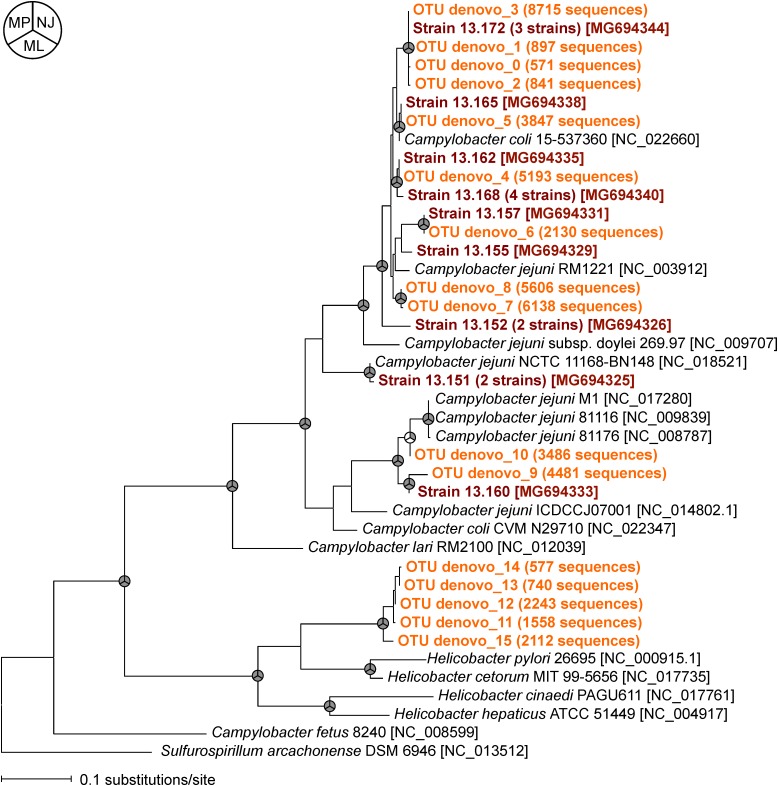
Phylogenetic relationships between *flaA* SVR sequences obtained by culture-dependent method (shown in brown) and those obtained by culture-independent MiSeq analysis (shown in orange). Phylogenetic tree constructed by maximum likelihood (ML) method is shown here, but similar trees were obtained by using neighbor-joining (NJ) and maximum parsimony (MP) methods. The nodes supported by bootstrap values (>70%) from 1000 replicates in the phylogenetic trees constructed by NJ, ML, and MP methods are indicated as gray in the pie charts next to the branches. The accession numbers of the reference strains in the DDBJ/EMBL/GenBank databases are indicated in brackets.

To compare the relatedness of the *flaA* of the isolated *Campylobacter* strains and those obtained by MiSeq, we constructed a phylogenetic tree (**Figure 3**). Almost all *flaA* genotypes obtained by culture-dependent method were also detected by culture-independent *flaA* SVR MiSeq sequencing, suggesting that the *flaA* SVR sequencing could be used to analyze the diversity of *Campylobacter* spp. without bacteria isolation. More *flaA* genotypes were identified by MiSeq probably because of the larger number of sequence reads in MiSeq. It is important to note that some *flaA* sequences obtained by MiSeq (five OTUs) were closely related to those of *Helicobacter* spp., which were not recovered by our isolation procedure.

## Discussion

Culture-independent *flaA* SVR sequencing could provide high-throughput information of *Campylobacter* diversity. To show this, we first designed a new primer set targeting *flaA* SVR to identify and differentiate *Campylobacter* strains, and compared the results with those obtained by a conventional DNA fingerprinting method. Then we applied the *flaA* SVR sequencing approach to analyze diversity of *Campylobacter* in geese fecal samples without bacteria isolation.

Similar degree of discrimination was identified between the *flaA* SVR sequencing and *flaA* RFLP methods. However, *flaA* RFLP could mistakenly assign some identical *Campylobacter* strains to a different group most likely due to insufficient fragment digestion. We also observed that some strains that were indistinguishable by *flaA* RFLP could be separated by *flaA* SVR sequencing. Our results were in agreement with previous comparative studies between DNA fingerprinting and sequencing genotyping methods. DNA sequencing methods such as those targeting *rpoB*, *slpA*, and *set* genes had higher reproducibility and higher discriminant power than DNA fingerprinting (Li et al., 2009). Therefore, the *flaA* SVR sequencing done with the new primers could be used as an alternative method to *flaA* RFLP to genotype *Campylobacter* strains.

The *flaA* SVR sequencing could be also used to analyze the diversity of *Campylobacter* spp. without bacteria isolation. For this purpose, we developed and optimized the sequence data analysis pipeline. Since our application purpose of *flaA* SVR sequencing was to identify genotypes of *Campylobacter* strains, not to compare *Campylobacter* populations among different samples, commonly used microbiome data analysis pipeline (e.g., Qiime; Caporaso et al., 2010) does not work well. We tried to recover as many genotypes (=OTUs) as possible, while removing genotypes generated by sequencing errors as much as possible. For this purpose, we used two parameters (*D* and *S*) to remove sequencing errors and identify OTUs. The optimized values for these parameters can vary by samples and target gene sequences. In our dataset, we identified that OTUs that contained >50 sequence reads (i.e., *D* = 50) at >98% alignment similarity (i.e., *S* = 98) represented >95% of all length-trimmed amplicon data (**Supplementary Table S2**). In general, increasing similarity value (*S*) provides increased number of OTUs; however, it also lowers the number of required duplicates (*D*) to represent >95% of all length-trimmed amplicon data. OTUs that contain small number of sequence reads (i.e., small *D* value) have higher chance of being generated by sequencing errors. Therefore, optimal *S* and *D* values should be determined based on the balance between resolution power and confidence required in a particular study.

Detection of same *flaA* genotype by both culture-dependent and -independent MiSeq approaches supports the usefulness of the *flaA* SVR sequencing for a rapid *Campylobacter* genotyping in environmental samples. Some *flaA* sequences obtained by MiSeq were closely related to those of *Helicobacter* spp. We designed a new primer set that can detect *flaA* SVR from *Campylobacter* spp. more broadly than the previous primers (Meinersmann et al., 1997), but it unexpectedly detected some *flaA* from *Helicobacter* spp. Our primers have several base mismatches to the *flaA* of *Helicobacter* spp. in the database; however, the database strains did not include *Helicobacter* strains isolated from geese. *Helicobacter* spp. were abundantly present in geese fecal samples (Green et al., 2012), and probably these *Helicobacter* strains may have *flaA* dissimilar to those of the database strains. However, it is unclear whether these geese-borne *Helicobacter* cause human diseases or not (Fox et al., 2006). Impact of these *Helicobacter* to public health should be investigated in the future.

In addition to the diversity information, we could also obtain quantitative data by using *flaA* primers for qPCR. Although PCR amplification efficiency was low with this *flaA* assay most likely due to relatively long amplicon size (*ca*. 400 bp), this could be overcome by using highly efficient DNA polymerases that are engineered or designed for long target amplification (e.g., KOD, Sso7d). Alternatively, quantification by digital PCR is independent of PCR amplification efficiency (Hindson et al., 2011), and therefore, can provide more accurate results of *flaA* copy numbers than conventional qPCR.

The density of *Campylobacter* in geese fecal samples as measured by *flaA* qPCR (4.06 log_10_ copies/g feces) was comparable to those reported previously (3.97 log_10_ copies/g feces) done with qPCR targeting *ciaB* of *C. jejuni* (Ishii et al., 2014a). There was disagreement between culture-dependent and culture-independent qPCR results. Samples positive for *Campylobacter* isolates were not always positives for *flaA* qPCR, and some samples negative for *Campylobacter* isolates showed positive results for *flaA* qPCR. This was probably due to (i) different detection sensitivity, (ii) detection of dead or VBNC cells by qPCR, and/or (ii) heterogeneous distribution of *Campylobacter* in the fecal samples. Culture-dependent method could detect 1 cell/g feces; while the quantification limit of our *flaA* assay was much higher (3 log_10_ copies/g feces). PCR-based methods could detect dead or dying cells. Although not tested in this study, use of propidium monoazide (PMA) could eliminate or significantly decrease PCR signals from dead *Campylobacter* cells (Castro et al., 2018), and therefore, could overcome this issue. We used 1 and 0.2 g of fecal samples for strain isolation and qPCR, respectively, and there is a possibility that *Campylobacter* was present only one of these subsamples.

While our culture-independent *flaA* SVR sequencing could provide high-throughput information of *Campylobacter* diversity in many environmental samples, there are several limitations. First, as mentioned above, DNA-based methods including *flaA* SVR sequencing could detect dead or dying cells. Use of PMA could overcome this issue and therefore should be tested in the future. Second, since *flaA* SVR sequencing method uses PCR, amplification biases could occur during PCR (Acinas et al., 2005). To minimize these effects, we carefully selected the PCR cycle numbers based on the qPCR results (Ishii et al., 2014b) and used a newly developed sequence analysis pipeline (Al-Ghalith et al., 2018). Lastly, PCR-based methods cannot provide information that isolated strains can do, such as pathogenic potential and antimicrobial resistance. One approach to assess potential health risks associated with *flaA* genotypes would be to use a comprehensive *flaA* database that includes clinical strains. Whole genomes of many clinical strains have been identified (Llarena et al., 2017), and therefore, it is possible to create such database. When *flaA* genotype highly similar to clinical strains is detected in environmental samples, it could pose greater health risks than other *flaA* genotypes. This approach should be examined in the future.

## Conclusion

This study shows that the culture-independent *flaA* SVR sequencing could provide high-throughput information of *Campylobacter* diversity in many environmental samples. By comparing *flaA* genotypes of environmental samples (e.g., water and food) and those of known animal sources, we could identify potential sources of *Campylobacter* in the environmental samples (Ishii, 2017). This kind of source tracking has been done using fecal indicator bacteria such as *E. coli* and *Bacteroides* (Ram et al., 2004; Unno et al., 2010), but has not been done targeting pathogens. This study was done to provide a tool for rapid *Campylobacter* source identification.

The same approach could be applied to study other pathogens. In addition, amplicon sequencing library preparation could be done in high throughput by using microfluidic qPCR format (Oshiki et al., 2018); therefore, source tracking of multiple pathogens could be done in a timely manner.

## Author Contributions

SI designed the research. MK, MM, and SI collected the samples. QZ, MK, TS, and MM performed the experiments. QZ, GA-G, MK, TS, DK, and SI analyzed the data. QZ and SI wrote the draft of the manuscript. All authors contributed to manuscript revision and read and approved the submitted version.

## Conflict of Interest Statement

The authors declare that the research was conducted in the absence of any commercial or financial relationships that could be construed as a potential conflict of interest.

## Footnotes

1 https://github.com/knights-lab/shi7/

2 http://nc2.neb.com/NEBcutter2/
