# Supplementary material for: High-Throughput flaA Short Variable Region Sequencing to Assess Campylobacter Diversity in Fecal Samples From Birds
Source: Front Microbiol. 2018 Sep 25;9:2201. doi: 10.3389/fmicb.2018.02201 (PMC6167966; doi:10.3389/fmicb.2018.02201)
Supplement: Supplementary file 1 [file Presentation_1.pdf]

Supporting Information

High-Throughput *flaA* Short Variable Region Sequencing to Assess *Campylobacter* Diversity in Fecal Samples from Birds

Qian Zhang[1], Gabriel A. Al-Ghalith[2], Mayumi Kobayashi[3], Takahiro Segawa[4,5], Mitsuto Maeda[3], Satoshi Okabe[3], D. Knights[1,2,6], and Satoshi Ishii[1,3,7,*]

[1]BioTechnology Institute, University of Minnesota, St. Paul, MN, USA

[2]Bioinformatics and Computational Biology, University of Minnesota, Minneapolis, MN, USA

[3]Division of Environmental Engineering, Graduate School of Engineering, Hokkaido University, Sapporo, Japan

[4]Center for Life Science Research, University of Yamanashi, Yamanashi, Japan

[5]National Institute of Polar Research, Tokyo, Japan

[6]Department of Computer Science and Engineering, University of Minnesota, Minneapolis, MN, USA

[7]Department of Soil, Water, and Climate, University of Minnesota, St. Paul, MN, USA

Pages: 6

Figures: 2

Tables: 2

*Correspondence: Satoshi Ishii, ishi0040@umn.edu.

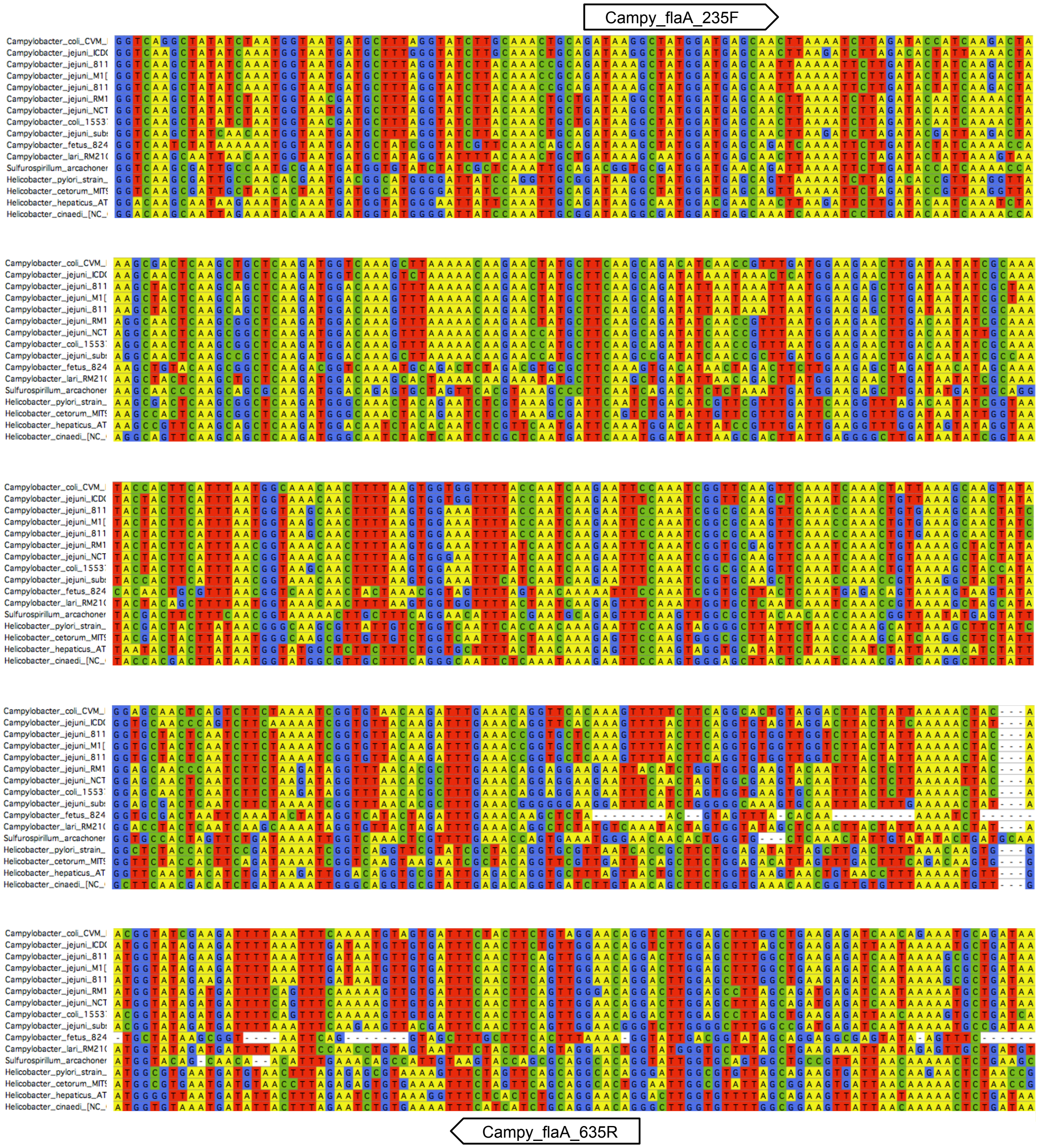

Figure S1. Annealing sites of the primers designed to amplify *flaA* from *Campylobacter* spp. Primer sequences are 5'-GATAARGCWATGGATGAGCA-3' for Campy_flaA_235F and 5'-CHGTYCCWACWGAAGTWGAA-3' for Campy_flaA_635R.

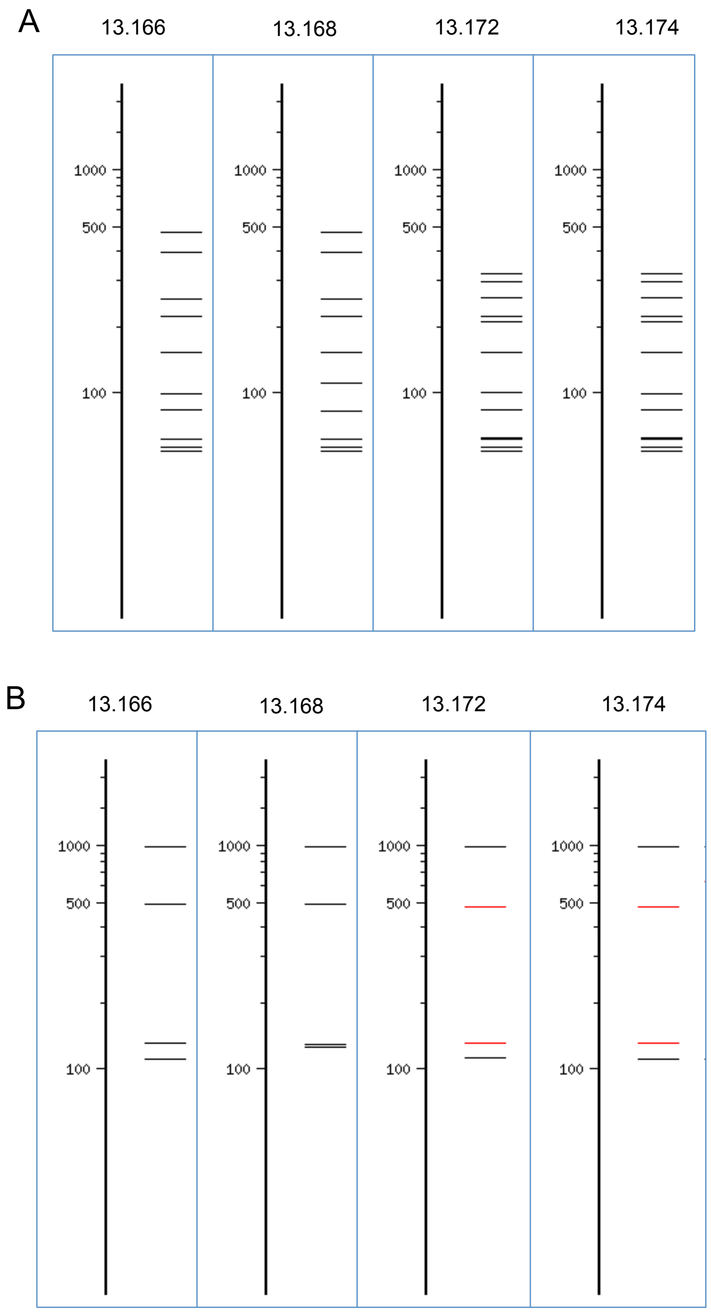

Figure S2. *In silico* digestion *flaA* amplicons with (A) *Dde* I and (B) *Hinf* I. *In silico* digestion was done using NEBcutter V2.0 software (http://nc2.neb.com/NEBcutter2/).

Table S1. Quantity of *flaA* measured by qPCR. Samples A–P and Q–Y were positive and negative, respectively, for *Campylobacter* strains. BDL, below detection limit (= 3 log_{10}/g feces)

Fecal sample ID		Quantity (log copies/g feces)
9/24/13	A	4.34
	B	3.65
	C	3.44
	D	BDL
	E	4.18
	F	4.76
10/4/13	G	BDL
	H	BDL
	I	BDL
	J	BDL
	K	BDL
	L	BDL
	M	BDL
	N	BDL
	O	BDL
	P	BDL
Samples negative for isolates	Q	4.49
	R	3.54
	S	3.57
	T	4.83
	U	4.61
	V	3.66
	W	3.50
	X	4.05
	Y	4.23

Table S2. Number of operational taxonomic units (OTUs), total number of sequence reads in OTUs, and coverage (%) as a function of number of duplicates (*D*) and sequence alignment similarity (*S*). We identified *D* = 50 and *S* = 98% as the optimal values to represent >95% of all length-trimmed *flaA* sequence data. Number of unique sequences after singleton removal was 51629, which was used as a denominator when calculating coverage values.

		Number of OTUs	Sequence alignment similarity (*S*)					
			97.00%		97.25%		97.50%	
			Total number of sequence reads in OTUs	Coverage (%)	Total number of sequence reads in OTUs	Coverage (%)	Total number of sequence reads in OTUs	Coverage (%)
Number of duplicates (*D*)	2	2108	51458	99.7	51367	99.5	51216	99.2
	3	971	51414	99.6	51310	99.4	51146	99.1
	5	473	51398	99.6	51284	99.3	51109	99.0
	10	186	51382	99.5	51261	99.3	51076	98.9
	25	46	51284	99.3	51101	99.0	50825	98.4
	50	16	51240	99.2	51044	98.9	50721	98.2
	100	11	51218	99.2	50994	98.8	50623	98.1

Table S2 (continued)

		Number of OTUs	Sequence alignment similarity (*S*)					
			97.75%		98.00%		98.25%	
			Total number of sequence reads in OTUs	Coverage (%)	Total number of sequence reads in OTUs	Coverage (%)	Total number of sequence reads in OTUs	Coverage (%)
Number of duplicates (*D*)	2	2108	50979	98.7	50553	97.9	49693	96.3
	3	971	50867	98.5	50371	97.6	49448	95.8
	5	473	50804	98.4	50251	97.3	49275	95.4
	10	186	50750	98.3	50141	97.1	49088	95.1
	25	46	50335	97.5	49481	95.8	48144	93.2
	50	16	50135	97.1	49135	95.2	47641	92.3
	100	11	49979	96.8	48875	94.7	47187	91.4

Table S2 (continued)

		Number of OTUs	Sequence alignment similarity (*S*)					
			98.50%		98.75%		99.00%	
			Total number of sequence reads in OTUs	Coverage (%)	Total number of sequence reads in OTUs	Coverage (%)	Total number of sequence reads in OTUs	Coverage (%)
Number of duplicates (*D*)	2	2108	48277	93.5	45731	88.6	41409	80.2
	3	971	47921	92.8	45166	87.5	40685	78.8
	5	473	47649	92.3	44721	86.6	39962	77.4
	10	186	47255	91.5	44019	85.3	38716	75.0
	25	46	45802	88.7	41929	81.2	35789	69.3
	50	16	44906	87.0	40662	78.8	34096	66.0
	100	11	44258	85.7	39816	77.1	33056	64.0
